# Major differences in crash carts for patient transport - a threat to patient safety

**DOI:** 10.1186/1757-7241-20-S2-P30

**Published:** 2012-04-16

**Authors:** Anders Møllekær, Bo Løfgren, Troels Krarup Hansen

**Affiliations:** 1 Research Center for Emergency Medicine, Aarhus University Hospital, Aarhus, Denmark; 2 Department of cardiology B, Aarhus University Hospital, Skejby, Aarhus, Denmark; 3 Department of Internal Medicine and Endocrinology, Aarhus University Hospital, Aarhus Sygehus, Aarhus, Denmark

## Background

Transporting critically ill patients between hospitals may be hazardous. Treatment of medical emergencies is paramount, and delivery of drugs during a medical emergency on patient transport may be lifesaving. Currently, there is no standardized crash cart and no guidelines on which drugs physicians should bring when escorting patients. There is increasing evidence that system differences may lead to human and adverse events, which may be fatal. The aim of this study was thus to examine the content and design of crash carts and furthermore, to investigate the knowledge of junior physicians on crash carts.

## Methods

All public hospitals in Central Region Denmark were contacted and the content and design of crash carts for inter-hospital patient transport were registered and systematically reviewed. Questionnaires on crash carts were handed out to junior physicians attending mandatory courses in Central Region Denmark from September to December 2010.

## Results

In total 7 hospitals had crash carts dedicated to inter-hospital transport and 8 different designs were identified. None of the 8 crash carts had identical medical content. Three crash carts had one or more drugs that were past the expiry date. A majority (75%) of crash carts did not contain drugs for treatment of anaphylactic shock. One crash card had almost identical labelling of drugs that made confusion between morphine and isoprenalin highly likely.

In total 116 (response rate 87%) questionnaires were answered. A majority (70%) of junior doctors were not introduced to the content of the crash cart prior to conducting their first patient transport. Half (47%) of junior physicians have performed an inter-hospital transport of an unstable patient and 75% of junior physicians have treated patients during transport.

## Conclusion

We demonstrate a pronounced diversity in crash carts content and design. Less than half of junior physicians were introduced to the content of the crash cart. Despite this, the majority had escorted an unstable patient and 75% had treated patients with drugs during transport. A standardized crash cart may strengthen knowledge of the content and reduce the risk of human error and adverse events.

